# Storytelling on Oral Grounds: Viewpoint Alignment and Perspective Taking in Narrative Discourse

**DOI:** 10.3389/fpsyg.2021.634930

**Published:** 2021-03-04

**Authors:** Kobie van Krieken, José Sanders

**Affiliations:** Centre for Language Studies, Radboud University Nijmegen, Nijmegen, Netherlands

**Keywords:** identification, narrative, perspective taking, storytelling, viewpoint alignment, interaction, conversation

## Abstract

In this paper, we seek to explain the power of perspective taking in narrative discourse by turning to research on the oral foundations of storytelling in human communication and language. We argue that narratives function through a central process of alignment between the viewpoints of narrator, hearer/reader, and character and develop an analytical framework that is capable of generating general claims about the processes and outcomes of narrative discourse while flexibly accounting for the great linguistic variability both across and within stories. The central propositions of this viewpoint alignment framework are that the distance between the viewpoints of participants in the narrative construal – narrator, character, reader – is dynamic and regulated by linguistic choices as well as contextual factors. Fundamentally, viewpoint alignment is grounded in oral narrative interaction and, from this conversation, transferred to the written narrative situation, varying between demonstration and invasion of the narrative subjects and guiding readers’ route of processing the narrative (*experiential* versus *reflective*). Our claim is that variations in viewpoint alignment are functional to the communicative context and intended outcomes of narratives. This is illustrated with the analysis of a corporate journalistic narrative that comprises both interactional and non-interactional aspects of storytelling. The concept of viewpoint alignment further explains the oral fundaments of narrative discourse in conversational storytelling and poses new questions on the relation between the dynamic processing of stories on the one hand and their static outcomes on the other.

## Introduction

A large part of human communication is inherently narrative, that is, representing specific events of specific persons in a specific spatiotemporal setting. Narrative styles and structures are ubiquitous in political communication (Shenhav, 2005; Polletta, 2009), journalism (Van Krieken, 2019), social media (Papacharissi and de Fatima Oliveira, 2012; Georgakopoulou, 2014), education (Devine et al., 2014), informal and professional interpersonal conversations (Avdi, 2008; Rühlemann, 2013), health communication (Wilkin and Ball-Rokeach, 2006), organizational discourse (Barker and Gower, 2010), and so on. In each of these communication subfields, research is carried out on both the processing and effects of narrative communication. A central finding is that narratives influence beliefs, attitudes, intentions, and behaviors more strongly than non-narratives (Braddock and Dillard, 2016). This impact can be explained by processes of “transportation” into the narrative world – the phenomenological experience of temporarily leaving the actual word behind and “traveling” to the story world (Gerrig, 1993) – and identification with narrative characters (see Van Laer et al., 2014, for a meta-analysis).

However, fundamental questions about what determines transportation, identification, and effective narrative communication have yet to be answered. Review studies on narratives in health communication and educational texts found numerous differences in the experimental narratives used as stimuli in effect studies, making it difficult to pinpoint the exact determinants of narrative persuasion (De Graaf et al., 2016; Sangers et al., 2020). Apart from great variation in length, modality, content, and context, which all inevitably influence the audience’s representation of a narrative, there are clues hinting at the importance of linguistic viewpoint phenomena. De Graaf et al.’s (2016) review study indicated that health narratives written from a first-person viewpoint tend to result in stronger identification and to be more effective than narratives written from a third-person viewpoint. However, a second-person viewpoint arouses more emotions and makes readers adopt the narrative character’s perspective more strongly than a first-person viewpoint does (Brunyé et al., 2009, 2011).

A different study found that variation in referential viewpoint affects identification: narrators who use pronominal references for narrative characters signal a proximate viewpoint, which can result in stronger identification than nominal references, by which narrators signal a more distant viewpoint (Van Krieken and Sanders, 2017). In addition, yet another study showed that the viewpoint marking by verbs of perception (e.g., “to see,” “to observe”) guides narrative readers into interpreting the story more strongly from the viewpoint of the character (rather than the narrator) than verbs without viewpoint marking qualities (Van Krieken, 2018). Finally, previous research found that readers are more strongly inclined to adopt a character’s perceptual perspective in present tense stories compared to past tense stories (Macrae, 2016): this indicates that the use of verb tense can guide readers into sharing viewpoints with the narrative character. Taken together, these studies signal that various linguistic manifestations of viewpoint – at least grammatical person, referential expressions, verbs of perception, and verb tense – appear to play a role in the cognitive representation and effect of narratives in their audiences.

The present paper builds on these signals and aims to transcend disciplinary boundaries by arguing that generally, narratives function through a central process of viewpoint alignment that can be traced back to oral storytelling. In conversations, narratives alternate between communicative participants who tell stories that function as entertaining, illustrative, and/or educational messages for the other(s) (Labov and Waletzky, 1967). The rhetorical point of stories is met by combining the representation of experience with reflection on experience; both involve viewpoint sharing, but in different ways. Essentially, we argue that the degree to which the viewpoints of participants in a given narrative context are aligned prompts different routes of processing – experiential versus reflective routes – and, by consequence, results in different outcomes. The theoretical model we develop in this paper contributes to our understanding of the language of perspective as grounded in interactive situations, and of its role in the narrative processes and effects which are grounded in oral storytelling patterns.

In the next section, we will first describe how viewpoint alignment is essential to conversational storytelling and how viewpoint in narrative discourse is linguistically varied throughout the story as it is brought by the narrator. Subsequently, we elaborate on functions of viewpoint alignment in terms of (a) the narrative styles, evoking acts, events, and sensations in readers; (b) the typical plot elements of the narrative; (c) the temporal pace of the narrative; (d) the routes of narrative processing; and (e) the different relations between character and reader therein. We argue that these functions of viewpoint alignment together show how narrative discourse is conceptually built on the oral speech situation, varying between demonstration and invasion of the narrative subjects and representing these subjects’ discourse from outside and within the narrative events.

In the third section, this framework will be illustrated by analyzing a narrative example; the analysis shows how variation in the viewpoint alignment serves different expected outcomes of the narrative in readers and relates these aims to traditional rhetorical functions of storytelling. In the final section, anchors for future experimental studies will be discussed that may empirically test the effect of viewpoint alignment on narrative processes and impact.

## Viewpoint Alignment Framework

### Joint Attention in Conversational Storytelling

Our point of departure is the consideration that human communication is fundamentally a matter of joint attention (Tomasello, 1995). Joint attention implies that two subjects align their viewpoints to jointly observe an “entity that they know they share” (Tomasello, 2008, p. 344), such as a third subject or an object, and in fact, the notion of viewpoint even presupposes the (actual or imagined) presence of this jointly observed entity (Tomasello, 2008). Joint attention skills develop early in childhood, in the first year of life, when infants start to direct and follow the visual attention of adults and, somewhat later, when they start participating in joint attention activities while acquiring their first words. Underlying the development of these skills is a child’s understanding that people are *intentional agents* who purposefully direct their own attention and that of the child to certain aspects of their mutual surroundings but not to other (Tomasello et al., 1993, p. 498). Human communication is similarly intentional in that we communicate to teach and learn, to share experiences, to construct identities, and to inform, convince, move, and entertain one another. From this perspective, viewing one another as intentional agents can be considered a prerequisite of successful human communication.

In establishing joint attention, people share viewing directions toward an object/subject or situation/event in their surroundings, cued by each other’s gestures and gaze directions (e.g., Mundy and Newell, 2007). This principle also applies to the narrative communication mode, with the crucial difference that in storytelling, the object/subject or situation/event of attention is generally not directly observable in the here-and-now communicative context but has to be imagined in a specific context (Van Krieken et al., 2017). Exactly this is what is challenging about viewing directions in narrative. In an oral conversational story, for example, a person may direct the other person’s attention to a specific setting as follows: “*I was at this beach party last week, and there was a huge bouncy castle with two kids jumping on it*. *They were actually jumping quite high. Then all of a sudden…*” The speaker may facilitate the establishment of joint attention and imagination with co-speech gestures, for example to indicate the size of the bouncy castle and the height of the jumps (Okada et al., 2013). Children acquire such gestures while narrating (Demir et al., 2015), but in written narratives, language is the only modality through which joint attention between the narrator and reader can be established. Either way, sharing viewing directions in narrative communication is primarily a cognitive act of viewing with “the mind’s eye” (cf. Green and Brock, 2002), guided by linguistic cues. The event-indexing model argues that this imaginary viewing requires the mental construction of a “microworld of what is conveyed in the story” and that this microworld consists of five dimensions that readers keep track of: time, space, protagonist, causality, and intentionality (Zwaan et al., 1995, p. 292). Upon construction of this mental representation of the story world, readers can vicariously experience the story events, becoming “immersed experiencers” (Zwaan, 2004, pp. 35–37). Immersive experiences have also been described as simulations, and mentally running simulations of story events and situations has been argued to enhance readers’ understanding of social life as well as their social cognitive skills (Mar and Oatley, 2008; Mar, 2018).

Once a cognitive representation of a story is established, readers can imaginatively view the story events from different angles. In this paper, we argue that the nature of this imaginary viewing can be both described and analyzed with the notion of *viewpoint alignment*. Viewpoint, or perspective, is deeply rooted in cognition as well as language and drives our bodily experiences of the world surrounding us and, as such, has strong explanatory force in understanding how human communication works. Yet, a theoretical framework that explicitly acknowledges the centrality of viewpoint in communication processes – and narrative processes in particular – and that explains these processes as a function of linguistic viewpoint phenomena remains to be developed. In the following, we propose that viewpoint alignment is a useful way of translating theoretical accounts of perspective that are developed in cognitive linguistics to the domain of narrative communication.

### Establishing and Varying Viewpoint in Narrative

The notion of viewpoint has received much attention in cognitive linguistics, a research domain aimed at illuminating the relation between the linguistic and the cognitive representation of objects, events, and situations. A central assumption is that this representation is dependent upon the viewpoint or vantage point, i.e., the (physical or metaphorical) point from which an object/subject or situation/event is represented (Langacker, 1987). For example, the viewpoint in an utterance like *Go to the shopping mall* is fundamentally different from the viewpoint in an alternative utterance like *Come to the shopping mall* (see Fillmore, 1966). In the first utterance, the speaker’s vantage point is positioned outside the shopping mall while in the second utterance it is positioned inside the shopping mall, implying that the speaker here-and-now is outside the shopping mall in the former versus inside in the latter. Similarly, the viewpoint of a sentence is typically located with the subject of that sentence rather than the object (Kuno, 1987). In the sentence *Amber called Matt*, the viewpoint is located with *Amber*, but in the passive equivalent *Matt was called by Amber*, the viewpoint is located with *Matt.* Note that different cues can have opposite effects, creating a complex viewpoint construal such as in *Her father called Amber.* Here, the grammatical viewpoint is juxtaposed to the referential situation: *her father* as description is semantically dependent on *Amber*. The possessive construction nuances the grammatical dominancy, profiles *Amber* as the main subject (semantically), and establishes the narrative viewpoint with her (Langacker, 1995). In short, the linguistic expression of an event affects hearers’ or readers’ cognitive construal of that event (Verhagen, 2007), and it also affects their comprehension of events in their discourse context (Black et al., 1979).

In narrative communication, the distance between the viewpoints of speaker/hearer and narrative characters is variable such that there may be more or less alignment. The scale can be considered to run from no viewpoint alignment at all at the one end to full viewpoint alignment at the other end, with different degrees of partial alignment in between. For instance, the narrative sentences *Amber went into the shopping mall* and *Amber came into the shopping mall* have different virtual vantage points, consequently including a viewing orientation outside the locker room versus an inside viewing orientation. In the cognitive representation of the virtual event, hearers are invited to join the narrative speaker (henceforth: narrator) in “viewing” along with Amber in the first sentence – to position themselves alongside Amber, going *into* a space of which there is no inside “view” provided – and from the front in the second – to “see” her entering *from* a space of which there is no inside “view” into a space in which hearers are imaginatively positioned themselves. As such, the first sentence (with *went*) invites the hearer to imagine that the narrator represents Amber’s viewing from up close, while the second (with *came*) presupposes a greater (virtual) distance; in fact, the narrator can invite hearers to represent, regulate, and alternate narrative characters’ viewpoints, using specific linguistic cues, even up to the point that represents no alignment. This is the case when narrator and character have different views on the narrative objects and events, like in an utterance such as *Amber had gone; she was not in the shopping mall.*

Typically, and opposite to a communicative speech situation here-and-now, where narrator and hearer may view a situation from different angles, the narrative communicative situation presupposes the conceptual alignment between the viewpoints of narrator and hearer/reader because the narrator is treated as a conversational participant (Bortolussi and Dixon, 2003): as such, the narrator provides the cognitive “filter” through which the hearer gains access to the narrative. As a result, the hearer shares the narrator’s view on the narrative objects and events as well as the narrator’s view on the character, while the character’s viewpoint and hence the character’s view on the narrative objects and events is inaccessible to the hearer. To the extreme, viewpoint alignment is achieved in case of a first-person-present-tense narrative construal: an “I” narration, or a direct quotation within a narrative. In nearly all narratives, such quotes will occur. Fundamental of all direct quotation in narrative discourse is the complete alignment of viewpoints between the narrator and the quoted character because in essence, quotes presuppose an embedded speech situation within the narrative (Sanders and Van Krieken, 2019). As such, they depict or represent an interaction and firmly ground the story in an – be it past or imaginative – oral conversational situation.

### Functions of Variation in Viewpoint Alignment

In most cases, the distance between the viewpoints of communicative participants in narrative discourse is variable and regulated by linguistic choices and contextual factors. As shown by the examples above, the process of viewpoint alignment is regulated by a range of linguistic phenomena. These include (but are not limited to) choices in grammatical construction, verb tense, expressions of perception and cognition, and choice of referential expressions (Van Krieken et al., 2017).^1^ These phenomena are indicative of variations in (a) narrative style, (b) narrative plot structure, (c) temporal pace, (d) narrative processing, and (e) narrator–character relation, which are each grounded in the interactive speech situation and which guide the readers’ representation of the narrative toward the intended outcomes of the narrative.

#### Narrative Styles: Demonstration and Invasion

The use of specific linguistic viewpoint markers results in a particular narrative style, corresponding with the particular viewpoint alignment position. A useful distinction is that between a *demonstrative* and an *invasive* style (Sanders, 2017). The demonstrative style that typically entails viewpoint nonalignment is characterized by an external view on a character and lacks internal viewpoint indicators. Instead, the character’s viewpoint is expressed by, for example, descriptions of the character’s acts and other events observable “from the outside” (cf. *landscape of action*, Bruner, 1990). An example is *Amber went to the shopping mall and spent the afternoon in the hardware store. In the evening, she had dinner with her father while discussing alternative ways to refurbish the bathroom*.

By contrast, the invasive style that typically entails viewpoint alignment is characterized by linguistic elements that provide access to the inner world of a narrative character (cf. *landscape of consciousness*, Bruner, 1990), such as verbs and adjectives referring to the character’s mind or emotions, thought reports, and stream of consciousness (Cohn, 1978; Van Krieken et al., 2017). An example is *Amber went to the shopping mall. Upon entering, she spotted a hardware store. It was difficult to find the hammer and the screwdriver she needed. That evening, she was proud to show her father the utensils she bought over dinner. Refurbishing the bathroom was her next goal.*

Quotes have different functions depending on the narrative’s style and can be distinguished in documenting versus dramatizing quotations (Van Krieken et al., 2016). Documenting direct quotes are typical for the demonstrative style: they *evaluate* events and situations within the story world from a narrative-external character viewpoint. An example is *Amber went to the shopping mall and spent quite some time. “I needed a hammer and a screwdriver, but it was not easy to find them”, she told her father*. Such quotes align the viewpoint of the narrator with the character’s viewpoint at a point in time *after* the narrative events, enhancing the reflective processing.

Dramatizing direct quotes, by contrast, are typical for the invasive style: they *mimic* events and situations within the story world from a narrative-internal character viewpoint (Van Krieken et al., 2016). An example is *Amber went to the shopping mall. Upon entering, she spotted a hardware store and started looking for the hammer and screwdriver she needed. “If they are not in this corner, where are they?” she wondered. “This is ridiculous. Why do they hide these things?” she spoke out loud.* Such quotes align the viewpoint of the narrator with the character’s viewpoint *during* the narrative events, enhancing the experiential processing.

#### Narrative Plot Structure: Climbing and Descending

Narratives may have a general demonstrative or invasive style, but more typically, narratives show a dynamic alternation between both styles in adjustment to the story structure and the progress of narrative time. Both in fictional and non-fictional narratives, the alignment between the viewpoints of narrator and character(s) is not fixed at all but may be aligned closer to and further away from one another from one narrative sentence to the next (Van Krieken et al., 2016). This alternation is enabled by the distinctive plot structure of narratives, which is inherently *temporal*. Time can be considered “the organizing axis” of storytelling (Neiger and Tenenboim-Weinblatt, 2016, p. 2). In producing and processing stories, narrative communicators move along this axis, thus traveling through narrative time (Sanders and Van Krieken, 2019).

Research on oral storytelling has clarified how different temporal frames serve different functions. Labov and Waletzky (1967) identified their basic story structure consisting of six elements: an *orientation* that paints the setting of the story in terms of time, place, and participants; *complicating actions* leading up to a *critical event* (the peak of the story), followed by a *resolution* (how the story ended) and *coda* (return from the story world to the here-and-now of the conversational setting). Finally, *evaluations*, often in the form of quotations, can be used throughout a story to express personal experiences and emotions related to the story events. Essentially, this pattern is chronological in nature, but variations in order may increase or release tension (Knobloch et al., 2004).

#### Temporal Pace: Acceleration and Deceleration

The progression of time in natural storytelling is subject to patterning of climbing and descending narrative peaks (Fleischman, 1990; Fludernik, 1991). Typically, temporal pace will decelerate upon the approach of narrative peaks and accelerate toward the end of plot structures, corresponding with viewpoint alignment variation. Deceleration and acceleration can be understood in terms of narrative duration: how is narrative time related to historical (story) time? Genette (1971) (p. 30) distinguishes between historical *récit*, which reduces the narrative duration compared to historical time (typical for plot elements such as complicating actions and resolution); dramatic *scene*, especially dialog, where narrative duration equals historical time (typical for plot elements such as critical event – peak – and evaluations); *stasis*, where narrative duration exceeds historical time (typical for plot elements such as complicating actions and evaluations); and *ellipsis*, which indicates historical time not covered in the narrative (typical for plot elements such as orientation and coda). Note that viewpoint alignment between narrator and character varies with these temporal frames: in case of *scene*, alignment is maximized, while it is reduced in *statis*, minimized in *récit*, and absent in *ellipsis*.

#### Narrative Processing: Reflective and Experiential

The way a story is structured in terms of plot structure and the corresponding temporal frames is functional to the intended processing of the story as well as the story’s intended outcomes (Sanders and Van Krieken, 2018) and is guided by the story’s style of narration. Crucial is the degree of alignment between the viewpoint of the character whose story is narrated in the story world, and the viewpoint of the hearers/readers (henceforth: readers) who imaginatively shift their viewpoint to the story world, guided by the speaker’s narration. When viewpoints are not aligned, such as in the demonstrative style “from the outside,” readers are led into a *reflective route*. Narratives, or parts thereof, that do not align the narrator’s viewpoint with the character’s viewpoint, offer readers a non-restricted view on the story world in which they are free to take a position at any point in narrative time, looking forward or backward, hence with room for own their viewpoint and for reflection; typically, a third-person past-tense narrative construal with a focus on story actions creates room for this reflective representation. Such a reflective route is expected to primarily evoke thoughts, evaluations, and considerations in readers.

In case of alignment between the viewpoints of narrator and character, such as in the invasive style “from the inside,” they move along the same path, and readers are invited to co-experience the story events as they unfold. Thus, the hearer’s “view” on the narrative is restricted, like a tunnel view: what is yet to be narrated can only be accessed and processed from the point of view of the character whose viewpoint is represented. Narrator and character thus share the same conception of past, present, and future as they jointly travel through the story world. A narrative construal with a focus on character sensations creates a limited representation in which the narrative time is experienced at the pace of the character’s experience. Consequently, the readers’ view of the narrative events, guided by the narrator, is restricted, and readers are thereby led into an *experiential route*. This route can be expected to primarily evoke sensations, feelings, and imaginations in readers (e.g., Zarantonello et al., 2013). Such experiential routing may be most functional around central peaks in storytelling, while reflective routing may be found in the lower peripheral areas of stories, and both routes are reflected in the relation between narrative characters and readers. Note that while these distinctions are conceptually sharp, actual narratives exhibit various combinations of the styles. In that sense, the distinctions might perhaps be said to represent opposite endpoints of a continuum.

#### Character and Reader: Spectating and Identifying

When viewpoints are not aligned, the nature of the mediated relation between the narrative character and the reader is mostly observatory: guided by the narrator, readers meet the character as spectators (see Oatley, 1999, p. 445). Spectatorship does not refer to the adoption of another person’s viewpoint but to a state in which readers relate their own viewpoint to the viewpoint of another person through observation. As Oatley (1999) (p. 445) puts it: “The reader becomes an unobserved observer in scenes of the lives of characters in the story world.” In the reflective style, narrative time may be stretched or, by contrast, compressed, such that the character’s experience of time is not guiding the representation, but a *reflection on* this experience in a slower or higher pace.

By contrast, when viewpoints are aligned, the nature of the mediated relation between the narrative character and the reader is one of *identification*. Rooted in psychological and developmental research, the original concept of identification refers to a process in which one temporarily sets aside his own viewpoint and imagines experiencing reality through someone else’s viewpoint (Freud, 1989 [1940]). In its original sense, then, identification is similar to viewpoint alignment. Throughout the years, the meaning of identification has been stretched – most notably by communication scholars – to include the audience’s adoption of a character’s emotions, attitudes, goals, and identity (e.g., Brown, 2015). This can be seen as the *result* of narrow viewpoint alignment: because the reader is “forced” to take on the viewpoint of the character, there is little to no room to *not* adopt the character’s emotions and attitudes.

## Viewpoint Alignment Analysis

In the following, we will demonstrate the premises of dynamic viewpoint alignment outlined above by applying this concept to a corporate journalistic narrative that has aspects of both spoken and written narrative and that exemplifies both demonstrative and invasive styles. In doing so, we show how a single story can evoke a processing route that is partly reflective and partly experiential and also how the framework can account for viewpoint alignment processes evoking various outcomes. We use a sample text, which is a narrative from a Dutch corporate context, the fire brigade, called *Fire & Fire Brigade*^2^. For the complete text (the original in Dutch and an English translation), please see the Appendix. Other aspects of this text are discussed in Van Krieken and Sanders (forthcoming).

### Interactive Story Context and Communicative Goals

Corporate journalistic media, such as *Fire & Fire Brigade*, are published by organizations to strengthen the commitment of staff to their organization and the quality of work in the organization by stimulating mutual communication (Van Ruler, 2005). Such magazines also serve, not infrequently, as a *public relations* means of communication for other stakeholders, such as the staff’s social context, but also governmental services, financiers, clients, and other relations, giving a clear picture of “what goes on” in the organization. It means that inherently, a *multiple audience design* is involved in corporate magazines; even if a corporate magazine is only distributed internally, it will always take into account any other potential reader groups.

The story central to this analysis is an edition of a recurring column, entitled *Under the Helmet.* The title expresses the idea that the column is about events of the uniformed fire brigade staff and also about the way these events are experienced. Such a column thus has two functions: firstly, to share the, at times, profound experiences of a part of the organization that fulfills a core function (in this case: fighting fires and saving people from dangerous situations) with other parts of the organization (in this case: office functions and other facilitating divisions of the fire brigade), and secondly, to show how such profound experiences are shared and processed. The storytelling itself also plays an exemplary role: talking about these experiences is permitted. The column deals with uniformed “operations” in which an active unit of the fire brigade is deployed because of an emergency situation; in this case, a young child that had become trapped in a fairground ride. The fire brigade’s task in such a case is to free the child as quickly and as unharmed as possible. Because of this urgency, in the setting of a public place with a great many bystanders, this apparently was a profound experience for the fire fighters involved. Sharing such an experience legitimizes the fire brigade as an organization: *this is what we do, this is what we are here for*. All employees and volunteers in the organization contribute from their own position to the performance of this core duty, and a story like this can strengthen their belief in the usefulness of their work. Of course, other reader groups such as the home front may also share in this legitimizing function. Importantly, such stories are implicitly educational about the moral frameworks within which this core duty is performed: *this is how we do this, and it’s the right way.* As such, the story is aiming at several interconnected communicative goals: informing (telling what the work entails); instructing (showing how to do the work); and emotionally and morally engaging (emotional engagement objectivemaking the work experiences tangible, and their importance evident).

### Viewpoint Alignment and Experience of Time

In this short story, the journalistic narrator firmly places the perspective with a single character, the experiencing subject, who is referred to in the third person: Klaas Wim Jansen^3^, first commanding officer of a unit that was involved in the operation concerned. The story is told almost completely chronologically by the narrator, who does not take part in the story and whose viewpoint alternatingly is not aligned, partially aligned, and completely aligned with the central character in this story. Parts in which Jansen is allowed to tell his story in direct quotes as an embedded narrator alternate with parts in which the journalistic narrator narrates about Jansen as a character in the main story; put in journalistic terms: Jansen is the source, who tells his story to a corporate journalist, who reports it on behalf of the organization. Jansen’s oral story is central to the text, and while it concerns his experiences, it is essentially characterized by its chronological structure (Labov and Waletzky, 1967). According to the Structural Affect Theory, this structure evokes more suspense than non-chronological story structures (Brewer and Lichtenstein, 1982). Empirical evidence shows that chronological structures do indeed generate suspense in readers (Knobloch et al., 2004) and also prompt stronger emotional reactions and enhance their experience of being transported into the story world (De Graaf and Hustinx, 2011). It can therefore be expected that readers of the fire brigade story experience a strong sense of transportation and suspense as the events unfold in the order in which they originally occurred to Jansen; thus, readers are invited to share Jansen’s viewpoint and emotions.

Linguistic devices such as verbs of perception enable the reader to see and experience the events through his eyes, for example: *When he hears en route that a child is trapped under a fairground attraction, it goes quiet in the fire engine.* Or: *At first, he doesn’t see the child.* Particularly in the present tense, verbs of perception as well as verbs of cognition are claimed to stimulate and guide readers’ identification with narrative characters by establishing a direct link with their minds (Damsteegt, 2005; Sanders, 2010), in this specific narrative: with Jansen. The narrator alternates such internal viewpoint representations with direct quotations, but not with stream of consciousness. In other words, it is an invasive story – readers imaginatively look through the eyes of the character – but by choosing to do this in the form of direct quotations, the story also has a demonstrative nature: everything is said and demonstrated clearly. Such alternation between invasion and demonstration can be considered characteristic of journalistic stories, which aim at engaging the audience while emphasizing the truthfulness and factuality of what is narrated (e.g., Van Krieken et al., 2016).

In all but one place in the story, the present tense is used by the narrator, which gives a film-like effect which serves this story well, since it is largely about a single exciting scene: the events appear immediately before the readers’ eyes, in a pace that is increasingly congruent to the lived events. The present tense has been found to facilitate identification (Macrae, 2016). Because the readers take in the experiences in the order in which Jansen had them, for the largest parts of the narrative, viewpoints are aligned – but not completely and not continuously. Both the past tense used in Jansen’s direct quotations, AND the 3^*rd*^ person perspective in the narrator’s parts indicate some distance between Jansen’s viewpoint and the reader’s. In the direct quotes the character is “*I*” but not *present*, and in the narrated utterances he is *present* but not “*I*.”

### Viewpoint Alignment and Direct Quotation

The direct quotations in this story do not only picture persons and their experiences in a lively way but also evaluate that which took place. In this story, the quotations express how grave, important, and profound these professional experiences were in the perception of those involved, as if it were something to learn from for future use (Boyd, 2009). Throughout the story, direct quotations are applied with two effects: building tension and transmitting emotions. The tension is visible at the beginning of the story: “*We responded and drove to the fairground, without me having any idea of what had happened.*” With this quote, the narrator invites readers to share the embedded narrator/character’s ignorance of the situation. *“‘I sympathize with you guys, lots of luck’, added the operator. Operators never say that. Afterwards, it turned out he’d seen images from a surveillance camera in the vicinity.”* Readers are thus stimulated by the narrator to share the viewpoint of Jansen in wondering what the operator knows that he does not yet know at this point in the story. Including the perspective of another character in the story, through direct quotations by the main character who himself quotes yet another person, helps building tension and attention. Thus, the chronology is rhetorically disturbed by combining a flash-forward and flashback, and a partial discrepancy in viewpoint alignment is established between the viewpoint of the narrator, the embedded narrator/character, and the reader. Quotations again are used to make the impact clear: *“I could see from the look on the traffic controller’s face (…) that the incident was a serious one.”*

All direct quotes are narrative-external, and the majority propels the narrative time in a reduced way such that the historical time exceeds the duration in the narrative (*récit*), for example in *“We responded and drove to the fairground (…)”*. This phrase represents Jansen as being quoted at a later point in time, looking back at acts and events at the narrative timeline. This is a construal typical for journalistic narratives, where it serves to legitimize the credibility of the narrative reconstruction on the basis of an interaction between journalist and source (Van Krieken et al., 2016). By contrast, narrative-internal quotes are evaluations and observation made at the time of the narrative that put the time to a halt (*scene*). In the sample narrative, few internal quotes are used and these are all embedded in external quotes, creating a multilayered temporal pace structure in which narrative time decelerates within an accelerated frame. This corresponds to a dynamic alternation between low and high viewpoint alignment. It is worth noticing that indirect representations are used when other characters than the embedded narrator are involved (“*Together with […] he decides that*” in the voice of the main narrator, “*Neither of us knew who she was*,” in the embedded narrator’s voice). In other words, only the main protagonist Jansen is represented with his own voice, which indicates that viewpoint alignment is shifting between these embedded (experiential, participating) and the external (reflective, journalistic) viewpoints.

Particularly interesting is the insertion of embedded narrative-internal quotes that express what *did not* happen but *could have happened*, not uncommon as a type of evaluation of the plot (Labov, 1997). The reflection of unreal versions of the plot puts the narrative time to a halt (*scene*) and enhances the critical point’s meaning. An example is provided by the underlined part of the quote in the following excerpt, which emphasizes the tension and risk: *“It wasn’t until we removed the covers from underneath the ride, that we could see properly how his little arm was stuck. This is never going to work, I thought. It looked really complicated”.* This present-tense quotation, embedded in an external quotation in the past tense, *shows*, rather than *tells*, how Jansen felt at the moment. Jansen introduces himself in the story as a thinking character, much like the inner thought in the story’s opening scene (*“‘Oh shit’, was my first thought”*). An additional function of such quotations is that readers can recognize themselves, due to the language use of the characters, who are representatives of the primary target grouptarget group, in the customary word choices and register of the quotations used. Notably, such quotes do not presuppose that the quoted material was uttered or thought in exact the same words. Clark and Gerrig (1990) explain that quotations are *non-serious* actions, that is, not actually occurring as such at the moment the quotation is uttered, but demonstrations of the particular narrative moment through selective depiction rather than description (Clark and Gerrig, 1990, p. 764). Here, these quotes are uttered at some point during the interaction of the source/character Jansen with the journalistic narrator.

Direct quotation in present tense, guiding toward complete viewpoint alignment, is typical for the story’s peak, in this case, the child’s liberation. The narrative prepares for the peak by quoting Jansen’s observations and emotions: *“When it was shouted downwards from above that the arm was loose, the relief was great. You really heave a sigh then.”* These utterances are remarkable in that they combine three linguistic viewpoint techniques: a physical demonstration, a de-activation of viewpoint, and a generic statement. In the first place, the character himself explains his position in the physical space at the exact moment of the liberation: people shouting down [implicitly: to where I was at that moment], from above. Such merging of viewpoints, representing the character’s physical position at the peak of the story, enables readers to share the experience of this crucial event with the character from the inside, as it were, and imagine it in an embodied way (Labov and Waletzky, 1967). This effect is established by the impersonal passive construction (*when it was shouted*) and nominalization (*the relief was great*), which, in combination, deactivate the potential viewpoints of other agents in the scene, inviting the reader to keep identifying with this character rather than with others (note that, stronger than in English, Dutch passive signals that the causer’s point of view should not be taken; see Cornelis, 1996, p. 247). To underscore the generalizability of the experienced feelings, the character evaluates the relief in a general expression: *“You really heave a sigh then.”* The use of the present tense and the generic “you” combine to give the statement a broader validity and express the fact that not only was the relief great but also it was an understandable and justified emotion, and that it is not surprising that anyone who goes through such an experience takes it personally (see also Van Krieken et al., 2015). Moreover, readers have been shown to internalize emotions more strongly when a second-person pronoun (rather than a first person pronoun) is used (Brunyé et al., 2011). Hence, an intense co-experience of Jansen’s relief is facilitated by the use of “you.” These combined techniques of impersonalization and generic “you” is in fact used several times in the story, as the following excerpt shows: “*Then there’s a commotion a bit further away […]. You’re just taken aback for a moment […]. Soon after that, all attention went back to the operation […].*” As such, this viewpoint deactivation seems to be indicative for the text’s narrative strategy as a whole.

### Viewpoint Alignment and Plot Structure

The analysis shows that the degree of viewpoint alignment may change during the story’s journey, such that narrative events in a given temporal frame may be processed differently than events in other frames. Fundamentally, the viewpoint alignment appears to be closer toward the core of the narrative, resulting in an experiential time conceptualization, whereas viewpoint alignment is less close at the beginning and end of the narrative, resulting in a distanced, or reflective, time conceptualization.

Accordingly, it appears that different temporal frames serve different functions. The *tellability* of the current story is determined by the uniqueness and impact of the events related: a child that is stuck in a machine, the tension that accompanies the rescue, and the vulnerable nature of the young victim give the story high attention value and offer many opportunities of empathizing with the events and persons involved. The story plot has a clearly recognizable basic structure, the chronological list of events starting with the *orientation*: the indication of the location and nature of a P1 report: Priority 1 (urgency). The events follow each other in quick succession: the unit deploys, is shown the way to the scene of the incident, arrives there, and assesses the situation: a child is trapped in a fairground ride. This problem is quite clearly the *complication*. With the help of various other units, the situation is analyzed, a plan of action is determined, and the child is freed, one step at a time. The *critical event* is when the fire brigade gets the child’s arm free. This moment of freeing merges into the *resolution* of the story: the child is transferred to the ambulance. The *resolution* for the fire brigade units involved is a different one: they go off duty, which means they are no longer on call, to evaluate what has happened; they subsequently go back on duty and complete their shift. The story ends with a *coda*: retelling the experience at home.

### Viewpoint Alignment and Narrative Theme

In this story, *looking* is a recurring theme, and crucial is looking at, and looking through the eyes of, the trapped child: *“(…) the look in his eyes spoke volumes. His eyes were screaming for help. Now and then he closed them (his eyes). When we started talking to him, he opened them and looked at us with that expression.”* This quotation occurs at a very compacted point in the time, in which the tension and emotion of the story come together without them having to be pointed out as such. Helpers and those helped look at each other repeatedly, and lengthily, and through this looking, there is a temporary but deeply felt contact, which expresses the great concern the professionals feel for the victim. In this particular part of the narrative, the narrator both invites and enables readers to look along – to literally align their viewpoint – with the main character and his colleagues, their attention drawn toward to the calm eyes of the child that form the focal point of the story. Such archetypal themes are universally human and make the story universally recognizable and profound (Campbell, 1949; Sanders and Van Krieken, 2019).

Notably, s*torytelling* itself is a recurring theme motive in the narrative analyzed in this article. First, during the evaluation at the fairground: *and you want to share your story.* Then: *The first two TS trucks and the HV go off duty, and all units return to the fire station for the evaluation. Everyone tells their story there again. When I got home, I told my story again.* The value being conferred here is that talking about the impressions and emotions that are unavoidable in such profound experiences is acceptable and recommendable. As such, the story, itself the *product* of storytelling in the organization, promotes storytelling as a *process*. Probably, in a traditionally masculine organization such as the fire brigade, talking about emotions arising from professional experiences is increasingly common. A corporate journalistic narrative like the current underlines this development as being good and desirable. The tellability of such stories is therefore also defined by their emotional and moral impact: telling people about that is worthwhile, much like conversational stories are being exchanged between people who want to share experiences in order to persuade each other of particular views on reality (Boyd, 2009).

### Viewpoint Alignment and Rhetorical Outcome

To conclude, our analysis has shown that demonstrative and invasive storytelling styles are combined and alternated according to the narrative’s plot structure and temporal frames, guiding readers closer to and then further away from characters in the story and finally bringing them to a moral conclusion. Thus, viewpoint alignment appears functional to different rhetorical outcomes of the story (Sanders and Van Krieken, 2018), with *phronesis* – i.e., “a form of moral sense making of the self that advances one’s practical wisdom and prudence” (Sanders and Van Krieken, 2018, p. 6) reflecting nonalignment, and *catharsis* – “deeply emotional experiences” (Sanders and Van Krieken, 2018, p. 13) – reflecting full alignment. More specifically, the main function of the experiential route is *pathos*, i.e., the reader’s empathizing with positive and negative feelings of positive expectation and agony (Kearney, 2007). These experiences are legitimized by evaluations and quotes that are fueled by *eleo*s or fear (Kearney, 2007), ensuring that these experiences are grounded and justified. On the peak of the narrative, physical experiences and positions are narrated in a merging style, functional to the story’s climax or *catharsis* in the reader’s experience of intense emotional relief (Koopman, 2013). The communicative goals of the narrative are advanced by these rhetorical functions: phronesis will help in informing about which complications the work entails, while pathos and eleos assist in instructing by showing how cautiously the work is done; and most importantly, catharsis serves to engage emotionally and morally with the work by making its experiences tangible, and their importance evident.

## Conclusion and Discussion

In this paper, we developed a model of viewpoint alignment to explain for the processes and outcomes of narratives that can be traced back to the oral origins of storytelling structure. In Table 1, an overview is generated of the categories we distinguished to explain the variation in viewpoint alignment. The four columns represent the two main modes of narration – demonstrating versus and invading the narrative subjects – and the two modes of direct quotation therein – representing the narrative subjects’ discourse from outside and within the narrative events – which, in various combinations and alterations, will dynamically guide the reader in reflective and experiential routes of narrative processing.

**TABLE 1:** Characteristics of different degrees of viewpoint alignment.

Degree of viewpoint alignment				
	− Spatiotemporal *distance* between viewpoints; object of conceptualization is viewed from *different* positions.	+/− Spatiotemporal *nearness* between viewpoints; object of conceptualization is viewed from a *similar* position.	+ Spatiotemporal *collapse* of viewpoints; object of conceptualization is viewed from one and the same position *outside* the narrative.	++ Spatiotemporal *collapse* of viewpoints; object of conceptualization is viewed from one and the same position *within* the narrative.

**Characteristics:**				
(a) Narrative style (Bruner, 1990; Van Krieken et al., 2016; Sanders, 2017)	Demonstrative narration of observed events (acts and situations)	Invasive narration of physical and psychological events (sensations, imaginations)	Documenting quotation (external direct quotes)	Dramatizing quotation (internal direct quotes)
(b) Narrative plot structure (Labov and Waletzky, 1967; Fludernik, 1991)	Orientation, coda	Complicating actions, resolution	Complicating actions, evaluations	Critical event (peak), evaluations
(c) Temporal pace (Genette, 1971)	Still/*Ellipsis*	Slower/*Récit*	Faster/*Stasis*	Realistic/*Scene*
(d) Processing route (Zarantonello et al., 2013)	Reflective	Experiential	Reflective	Experiential
(e) Mediated relation between character and reader (Oatley, 1999; Van Krieken et al., 2016)	Spectatorship/distant	Identification/close	Legitimizing/distant	Merging/close
(f) Expected rhetorical outcome (Kearney, 2007; Sanders and Van Krieken, 2018)	*Phronesis*	*Pathos*	*Eleos*	*Catharsis*

As conceptual model, viewpoint alignment is intended to complement influential theories of narrative communication, such as the Entertainment-Education and Elaboration Likelihood Model (Slater and Rouner, 2002), the Transportation-Imagery Model (Green and Brock, 2002), and the Mental Models approach (Busselle and Bilandzic, 2009). Even though it may not be explicitly acknowledged in these theories, the notion of viewpoint alignment is capable of explaining and predicting the mechanisms they describe. For instance, fundamental to the Transportation-Imagery Model and Mental Model approaches is the projection of the reader’s viewpoint into the story world as a prerequisite for their engagement with the story characters and events and their sense of “getting lost” in the story. In a different way, the “central” processing route as distinguished in the Entertainment-Education and Elaboration Likelihood Model implies more room for one’s own viewpoint in the narrative processing compared to the “peripheral route.” Importantly, our model explains how such narrative processes are guided by linguistic structures that regulate the degree of viewpoint alignment.

Our model is furthermore distinct from previously developed theories in its assumptions about the nature of narrative processes. First, the Transportation-Imagery Model describes narrative processing as a process different from central and peripheral processes, but in our approach, experiential and reflective routes of (narrative) communication can be seen as two *types* of narrative processing routes. Second, the viewpoint alignment approach furthermore does not assume that there are differences in *strength* or *depth* of processing of the different routes (contrary to the assumptions of the Entertainment-Education Elaboration Likelihood Model). Experiential and reflective routes could both be equally intense. Crucially, both types are not mutually exclusive but can complement one another, as we have posited in our analysis of the corporate narrative. In our view, this is an essential assumption in understanding the range of outcomes a narrative can have (e.g., on the reader’s beliefs, attitudes, knowledge, and behavior) and how such static outcomes are the result of a dynamic process.

Questions about the relation between the dynamic processing of narrative discourse and the static outcomes could be answered in future experimental studies. The design of such studies can be informed by the narrative characteristics summarized in Table 1. For example, by manipulating the characteristics of demonstrative narrating, invasive narrating, and direct quotations, studies could test their effect on readers’ degree of identification with the narrative character and the potential subsequent persuasive impact of the story. The use of online measures to assess readers’ identification with the character, such as fMRI or galvanic skin responses (Van Krieken et al., 2017), could help to establish the degree to which this process varies during reading and to pinpoint the precise linguistic elements that increase (or decrease) identification. Studies in this direction are essential to test the expectations of our viewpoint alignment framework and to advance our understanding of the function and impact of perspective and perspective-taking in the language of stories.

At a conceptual level, the model developed in this paper supports the notion that time and perspective are intricately connected in the representation of narratives (see Van Krieken et al., 2019). Our model fleshes this idea further out by proposing that this connection, too, is dynamic by nature and develops over the course of a narrative: narrative time can be presented in a condensed manner in a given part of the story but in an expanded way in another part, which is intrinsically related to a change in the degree of viewpoint alignment. This conceptualization is firmly rooted in oral storytelling patterns and helps to understand how narratives can fulfill multiple communicative goals at the same time, which is often the case for nonliterary narratives such as corporate stories. Understanding narrative processing as a dynamic alignment of viewpoints that alternately invite readers to experience or reflect on the story events, explains how a single story can inform, instruct, move, persuade, and empower readers all at once.

## Data Availability Statement

The original contributions presented in the study are included in the article/supplementary material, further inquiries can be directed to the corresponding author/s.

## Author Contributions

KK and JS contributed equally to the manuscript and contributed to introduction, framework, analysis, conclusion, and discussion. Both authors contributed to the article and approved the submitted version.

## Conflict of Interest

The authors declare that the research was conducted in the absence of any commercial or financial relationships that could be construed as a potential conflict of interest.

## Footnotes

1 In oral and (audio)visual narratives, the degree of viewpoint alignment is also regulated by extralinguistic phenomena, such as gesture and eye gaze (in spoken narratives; Parrill, 2010; Sweetser and Stec, 2016), camera positions (in filmic narratives; Alber, 2017), and pictographic cues (in comics; Borkent, 2017).

2 https://issuu.com/sduuitgeversbrandbrandweer/docs/bb201710, p. 31 (accessed January 24, 2021).

3 For reasons of privacy, we use a pseudonym for the officer’s real name.

## Appendix

Text from Dutch corporate magazine Brand & Brandweer [Fire & Fire Brigade], in particular a column in the series Onder de helm [Under the Helmet], entitled Ik leef met jullie mee, veel sterkte! [I Sympathize with You Guys, Lots of Luck]. The text was written by Jildou Visser and published in October 2017.

Original Dutch version:

P1 letsel ijssalon Intermezzo Heuvelring Tilburg. Voor die melding wordt eerste bevelvoerder Klaas Wim Jansen op 22 juli gealarmeerd. ‘We rukten uit naar de kermis, maar ik had nog geen idee wat er aan de hand was.’ Als hij aanrijdend hoort dat een kind bekneld ligt onder een kermisattractie, wordt het stil in de TS. Jansen: ‘Ow shit, was het eerste dat door mijn hoofd schoot. “Ik leef met jullie mee, veel sterkte jongens”, zei de centralist nog. Dat zegt een centralist nooit. Achteraf bleek dat hij beelden van een bewakingscamera in de buurt had gezien.’

De brandweerlieden worden bij de kermis opgewacht door een verkeersregelaar. ‘Aan de blik op het gezicht van de verkeersregelaar en de manier waarop hij ons zwaaiend de weg wees, zag ik dat het een ernstig incident was. Vervolgens ging het zo snel. Vijftig meter verder waren we er al. Er waren veel mensen, schreeuwend, in paniek.’ In eerste instantie ziet Jansen het kind niet. Door omstanders wordt hij erop gewezen dat het slachtoffertje op ongeveer twee meter hoogte ligt, bekneld onder een karretje van de kermisattractie. ‘Dat zag er niet goed uit.’ De attractie voor kleine kinderen bestaat uit aaneengeschakelde karretjes die langzaam omhoog en omlaag rijden. ‘Het jongetje was uit een karretje gevallen en eronder terechtgekomen. Omstanders tilden het wagentje al op, zodat het niet meer met het volle gewicht op zijn buik lag. Hij was bij kennis en erg rustig. Hij heeft tijdens de hele inzet niet één keer geschreeuwd, maar die blik in zijn ogen zei alles. Zijn ogen schreeuwden om hulp. Af en toe sloot hij z’n ogen. Als we tegen hem begonnen te praten, deed hij z’n ogen weer even open en dan keek hij ons aan met die blik.’

Op dat moment weten de brandweerlieden nog niet hoe het jongetje vastzit. De bemanning van de eerste TS stabiliseert het karretje, haalt het daarna los en verwijdert het. Pas dan zien de manschappen dat de arm van de jongen klem zit door de sleuf met het mechanisme waarmee de karretjes werden voortbewogen. ‘Toen we de kappen aan de onderkant van de attractie weghaalden, konden we pas echt goed zien hoe het armpje vastzat. Dit komt nooit meer goed, dacht ik. Het zag er erg ingewikkeld uit’, aldus Jansen. Samen met de bevelvoerders van de tweede TS en het HV besluit hij dat ze de reling met de verlichting doorknippen om beter zicht te krijgen. Op dat moment keert een omstander zich tegen de brandweer. ‘Een vrouw liep naar een van mijn manschappen toe toen hij de reling door wilde knippen. Hij keek mij aan. We wisten beiden niet wie het was. Het had een bezorgde ouder kunnen zijn, maar toen ze aan mijn manschap begon te trekken, wisten we genoeg. De politie greep gelukkig snel in’, vertelt Jansen. ‘Dan ontstaat even verderop een opstootje als een tweede persoon zich tegen de politie keert. Je bent even heel verbaasd, omdat je probeert een kind te redden en dan zo wordt tegengewerkt. Snel daarna ging de focus weer volledig op de inzet en op het kind terwijl de politie het vechtpartijtje verderop afhandelde.’

Nadat de reling kapot is geknipt, buigen de brandweerlieden de stalen plaat om zodat ze beter kunnen zien hoe de constructie in elkaar zit. Jansen: ‘Daarna hebben we stukje voor stukje in de attractie geknipt om het mechanisme waar de jongen in vastzat los te maken. Toen van bovenaf naar beneden werd geroepen dat de arm los was, was de opluchting groot. Dan slaak je wel even een zucht.’ Enkele manschappen helpen de ambulancemedewerkers om de jongen naar beneden te tillen. ‘Daarna hebben we op de kermis direct een eerste nagesprek gehouden. Het was voor ons allemaal een flinke klus, dan wil je je verhaal wel even kwijt.’ De eerste twee TS’en en de HV gaan buiten dienst en alle eenheden keren daarna terug naar de kazerne voor de evaluatie. Daar doet iedereen nogmaals zijn verhaal. Na de evaluatie besluit de ploeg weer in dienst te gaan. ‘We hebben die dag nog een paar meldingen gehad. Dat was goed. Het is fijn om wat afleiding te hebben.’ Na zijn dienst keert Jansen naar huis. ‘Thuis heb ik mijn verhaal nog een keer gedaan. Ik praat normaal nooit over inzetten, maar deze inzet was bijzonder en vergeet ik nooit meer. Het is fijn als er dan thuis een luisterend oor is.’

English translation:

P1 injury Intermezzo ice cream parlor, Heuvelring in Tilburg. Commanding officer Klaas Wim Jansen was the first to receive this report on 22 July. “We responded and drove to the fairground, without me having any idea of what had happened.” When he hears en route that a child is trapped under a fairground attraction, it goes quiet in the fire engine. Jansen: “‘Oh shit’ was my first thought. ‘I sympathize with you guys, lots of luck’, added the operator. Operators never say that. Afterwards, it turned out he’d seen images from a surveillance camera in the vicinity.”

The fire fighters are met at the fairground by a traffic controller. “I could see from the look on the traffic controller’s face, and the way he waved us through, that the incident was a serious one. After that, everything went so quickly. Fifty meters further, and we’d arrived. There were lots of people, screaming in panic.” At first, Jansen doesn’t see the child. He is pointed out by bystanders that the victim is about two meters high, trapped under a trolley of the fairground attraction. “It didn’t look good.” The ride, for small children, consists of a row of cars, linked together, that ride slowly upward and downward. “The little boy had fallen out of a ride car and landed underneath it. Bystanders were already lifting the ride car, so the full weight of it wasn’t on his stomach any more. He was conscious and very calm. He hasn’t screamed once during the whole operation, but the look in his eyes spoke volumes. His eyes were screaming for help. Now and then he closed his eyes. When we started talking to him, he briefly opened his eyes again and looked at us with that expression.”

At that point, the fire fighters don’t know how exactly the little boy is trapped. The crew of the first TS truck stabilizes the ride car, then disconnects and removes it. Only then do they see that the little boy’s arm is jammed in the slit of the mechanism that moved the cars forward. “It wasn’t until we removed the covers from underneath the ride, that we could see properly how his little arm was stuck. This is never going to work, I thought. It looked really complicated”, according to Jansen. He and the commanding officers of the second fire truck and the other emergency vehicle decide to start by cutting through the railing with the lighting to get a better view. At that point, a bystander turns against the fire fighters. “A woman walked toward one of my crew just as he was about to cut through the railing. He looked at me. Neither of us knew who she was. She might have been a concerned parent, but when she started pulling at my crew member, it was clear. Luckily, the police intervened quickly”, Jansen tells. “Then there’s a commotion a bit further away, when a second person turns on the police. You’re just taken aback for a moment, because you’re trying to save a child and people are turning on you like that. Soon after that, all attention went back to the operation and the child, while the police dealt with the fight further along.”

Once the railing is cut, the fire fighters bend the steel plate, so they have a better view of the construction of the ride. Jansen: “After that, we have cut through the ride, a bit at a time, to free the mechanism the boy was stuck in. When it was shouted downward from above that the arm was loose, the relief was great. You really heave a sigh then.” A few crew members help the ambulance staff lift the boy down. “Afterward, we immediately had an initial meeting about the operation, there at the fairground. It was a big operation for all of us, so you want to talk about it.” The first two TS trucks and the HV go off duty, and all units return to the fire station for the evaluation. Everyone tells their story there again. Following the evaluation, the crews decide to go back on duty. “We have been called out a few more times that day. That was fine. It’s good to have some distraction.” After his shift, Jansen returns home. “At home, I have told my story again. I don’t usually talk about operations, but this one was special, and I’ll never forget it. It’s nice when there’s someone at home to talk to then.”
